# Biologic Therapy in Refractory Non-Multiple Sclerosis Optic Neuritis Isolated or Associated to Immune-Mediated Inflammatory Diseases. A Multicenter Study

**DOI:** 10.3390/jcm9082608

**Published:** 2020-08-11

**Authors:** Alba Herrero-Morant, Carmen Álvarez-Reguera, José L. Martín-Varillas, Vanesa Calvo-Río, Alfonso Casado, Diana Prieto-Peña, Belén Atienza-Mateo, Olga Maiz-Alonso, Ana Blanco, Esther Vicente, Íñigo Rúa-Figueroa, Laura Cáceres-Martin, José L. García-Serrano, José Luis Callejas-Rubio, Norberto Ortego-Centeno, Javier Narváez, Susana Romero-Yuste, Julio Sánchez, Paula Estrada, Rosalía Demetrio-Pablo, David Martínez-López, Santos Castañeda, José L. Hernández, Miguel Á. González-Gay, Ricardo Blanco

**Affiliations:** 1 Rheumatology, Ophtalmology and Internal Medicine, Hospital Universitario Marqués de Valdecilla, Av. de Valdecilla, 25, 39008 Santander, Spain; alba.herrero@scsalud.es (A.H.-M.); carmen.alvarezr@scsalud.es (C.Á.-R.); vanesa.calvo@scsalud.es (V.C.-R.); alfonso.casado@scsalud.es (A.C.); diana.prieto.pena@gmail.com (D.P.-P.); belen.atienza@scsalud.es (B.A.-M.); rosalia.demetrio@scsalud.es (R.D.-P.); david.martinez@scsalud.es (D.M.-L.); 2 Rheumatology, Hospital Sierrallana, Barrio Ganzo, s/n, 39300 Torrelavega, Spain; jlmvarillas@gmail.com; 3 Rheumatology and Ophtalmology, Hospital de Donostia, Paseo Dr. Begiristain, 117, 20080 Donostia, Spain; OLGA.MAIZALONSO@osakidetza.eus (O.M.-A.); ablancoesteban@hotmail.com (A.B.); 4 Rheumatology, Hospital Universitario de La Princesa, C/Diego de León, 62, 28006 Madrid, Spain; efvicenter@gmail.com (E.V.); scastas@gmail.com (S.C.); 5 Rheumatology, Hospital Universitario de Gran Canaria Doctor Negrín, C/Plaza Barranco de la Ballena, s/n, 35010 Las Palmas de Gran Canaria, Spain; iruafer@gobiernodecanarias.org (Í.R.-F.); lcmlcm@hotmail.com (L.C.-M.); 6 Internal Medicine and Ophtalmology, Hospital San Cecilio, Av. del Conocimiento, s/n, 18016 Granada, Spain; w7461322@ugr.es (J.L.G.-S.); jlcalleja@telefonica.net (J.L.C.-R.); nortergo@gmail.com (N.O.-C.); 7 Rheumatology, Hospital de Bellvitge, Carrer de la Feixa Llarga, s/n, 08907 L’Hospitalet de Llobregat, Spain; fjnarvaez@bellvitgehospital.cat; 8 Rheumatology, Complejo Hospitalario Universitario de Pontevedra, Loureiro Crespo, 2, 36002 Pontevedra, Spain; susanaromeroyuste@gmail.com; 9 Rheumatology, Hospital Universitario 12 de Octubre, Av. de Córdoba, s/n, 28041 Madrid, Spain; jsm132@hotmail.com; 10 Rheumatology, Hospital de Sant Joan Despí Moisès Broggi, Carrer de Jacint Verdaguer, 90, 08970 Sant Joan Despí, Spain; paulavestradaa@gmail.com

**Keywords:** optic neuritis, biologic therapy, rituximab, tocilizumab, adalimumab, infliximab

## Abstract

We aimed to assess the efficacy of biologic therapy in refractory non-Multiple Sclerosis (MS) Optic Neuritis (ON), a condition more infrequent, chronic and severe than MS ON. This was an open-label multicenter study of patients with non-MS ON refractory to systemic corticosteroids and at least one conventional immunosuppressive drug. The main outcomes were Best Corrected Visual Acuity (BCVA) and both Macular Thickness (MT) and Retinal Nerve Fiber Layer (RNFL) using Optical Coherence Tomography (OCT). These outcome variables were assessed at baseline, 1 week, and 1, 3, 6 and 12 months after biologic therapy initiation. Remission was defined as the absence of ON symptoms and signs that lasted longer than 24 h, with or without an associated new lesion on magnetic resonance imaging with gadolinium contrast agents for at least 3 months. We studied 19 patients (11 women/8 men; mean age, 34.8 ± 13.9 years). The underlying diseases were Bechet’s disease (*n* = 5), neuromyelitis optica (*n* = 3), systemic lupus erythematosus (*n* = 2), sarcoidosis (*n* = 1), relapsing polychondritis (*n* = 1) and anti-neutrophil cytoplasmic antibody -associated vasculitis (*n* = 1). It was idiopathic in 6 patients. The first biologic agent used in each patient was: adalimumab (*n* = 6), rituximab (*n* = 6), infliximab (*n* = 5) and tocilizumab (*n* = 2). A second immunosuppressive drug was simultaneously used in 11 patients: methotrexate (*n* = 11), azathioprine (*n* = 2), mycophenolate mofetil (*n* = 1) and hydroxychloroquine (*n* = 1). Improvement of the main outcomes was observed after 1 year of therapy when compared with baseline data: mean ± SD BCVA (0.8 ± 0.3 LogMAR vs. 0.6 ± 0.3 LogMAR; *p* = 0.03), mean ± SD RNFL (190.5 ± 175.4 μm vs. 183.4 ± 139.5 μm; *p* = 0.02), mean ± SD MT (270.7 ± 23.2 μm vs. 369.6 ± 137.4 μm; *p* = 0.03). Besides, the median (IQR) prednisone-dose was also reduced from 40 (10–61.5) mg/day at baseline to. 2.5 (0–5) mg/day after one year of follow-up; *p* = 0.001. After a mean ± SD follow-up of 35 months, 15 patients (78.9%) achieved ocular remission, and 2 (10.5%) experienced severe adverse events. Biologic therapy is effective in patients with refractory non-MS ON.

## 1. Introduction

Optic neuritis (ON) is an acute inflammatory optic neuropathy that may be associated with dramatic visual loss and an important decrease in quality of life in absence of an adequate treatment. Multiple Sclerosis (MS) ON, the most common form of presentation, is characterized by unilateral acute retroocular pain and visual loss, more commonly observed in Caucasian women between 18 and 50 years [1]. Visual acuity (VA) in patients with MS-ON usually improves within a few months even without treatment [2,3,4]. Non-MS ON is less frequent and can be an isolated disorder or related to infections and immune-mediated diseases such as Neuromyelitis Optica (NMO) or other systemic diseases [5]. Non-MS ON may have atypical features such as male gender, age less than 18 or greater than 50 years, absence of pain and bilateral presentation [5]. In non-MS ON, a chronic progressive disease is more common. Flare-ups are frequent, leading often to visual loss [3,6]. If not promptly treated, the visual outcome can be devastating, causing a severe visual loss, and even with adequate treatment, many patients may worsen over months [7,8,9,10].

Therapy has been mostly focused on MS-ON. According to the Optic Neuritis Treatment Trial [2], in patients with MS or isolated ON, intravenous (i.v.) high-dose glucocorticoids followed by oral prednisolone may accelerate the visual recovery. Nevertheless, there is not a significant improvement of VA at 6 months and 1 year compared to placebo. The most recent Cochrane Review identified six randomized controlled trials with a total of 750 participants. Five trials (*n* = 633) only analyzed MS or isolated ON. It concluded that there is still no definitive evidence that i.v. glucocorticoids improve visual outcomes after 6 months of treatment [11].

Non-MS ON treatment has been less frequently assessed. Glucocorticoids, plasmapheresis and intravenous immunoglobulins may be effective in acute attacks, particularly in NMO [12,13,14,15]. Three recent clinical trials have analyzed the use of satralizumab, eculizumab and inebilizumab in NMO [16,17,18]. All three have demonstrated a reduction of risk of NMO attack compared to placebo. Conventional immunosuppressive therapies have demonstrated clinical benefits for reducing relapses [6], but biologic agents have been rarely used. Thus, rituximab (RTX), an anti-CD20 monoclonal antibody, tocilizumab (TCZ), an IL-6 monoclonal antibody [12,19,20,21,22], and anti-TNFα therapy, especially adalimumab (ADA) and infliximab (IFX), have been only used in some refractory cases [23,24,25,26,27].

Taking into account all these considerations, this study aimed to assess the efficacy and safety of biologic therapy in refractory non-MS ON, both isolated and associated with immune-mediated inflammatory diseases.

## 2. Experimental Section

### 2.1. Design and Enrollment Criteria

We performed an observational open-label multicenter study that included 19 patients diagnosed with non-MS ON refractory to systemic glucocorticoids and at least one conventional immunosuppressive drug.

Patients were diagnosed with non-MS ON at the Ophthalmology, Neurology and Rheumatology Units of eleven different referral Spanish Hospitals. Since biologic therapy is an off-label indication for ON, written informed consent was requested and obtained from all the patients. The study was approved by the Clinical Research Ethics Committee (ethical approval code: 2020.010).

Diagnosis of ON was based on clinical features, ophthalmologic examination, high-definition optical coherence tomography (OCT), magnetic resonance imaging (MRI) and cerebrospinal fluid analysis (CSF). The presence of subacute vision loss in adults, along with a relative afferent papillary defect (RAPD) was required for diagnosis [2,3,4,5]. In addition, MRI findings such either T1-weighted gadolinium enhancement of the optic nerve, or T2-weighted optic nerve hyperintensity were needed for diagnosis [28,29]. Aquaporin-4 water channels -IgG and Myelin Oligodendrocyte Glycoprotein -IgG were assessed in all patients. Both unilateral and bilateral cases of ON were included in the diagnosis.

Inclusion criteria were as follows: (a) non-MS ON, (b) lack of response to previous treatment with a high dose of systemic glucocorticoids defined as more than 7.5 mg/day for more than 3 months and (c) to at least one conventional immunosuppressive drug at its standard doses.

MS was excluded by the McDonald’s criteria that were based on clinical, imaging and laboratory parameters [30].

As indicated by the Spanish Biologic Treatment Administration National Recommendations, the presence of infectious diseases had to be ruled out before starting the biologic treatment. To exclude latent tuberculosis, a tuberculin skin testing (PPD) and/or an interferon assay (quantiFERON) and chest radiography were performed. In positive cases, prophylaxis with isoniazid was initiated for at least 4 weeks before using the biologic treatment and was maintained for 9 months. The presence of malignancies was excluded in all patients [31,32,33,34,35,36,37,38,39].

According to current guidelines, avoiding the use of anti-TNFα drugs is recommended in patients with a history or familiar occurrence of demyelinating diseases [4,40]. Besides, a relatively common anti-TNFα Adverse Event (AE) is ON [41,42]. Thus, anti-TNFα therapy was avoided in NMO (demyelinating disease).

### 2.2. Outcome Variables

The outcome variables were the efficacy and the safety of biologic therapy. The main outcomes of efficacy were best-corrected visual acuity (BCVA), retinal nerve fiber layer (RNFL), and macular thickness (MT). Secondary outcomes were remission, number of relapses and sparing effect of glucocorticoids. To determine safety, AE were evaluated.

BCVA was estimated by the logMAR chart. The optic nerve was evaluated measuring with an OCT the loss of retinal nerve fibers with RNFL analysis. The loss of retinal nerve fibers associated with optic atrophy in patients with optic neuropathies can easily be visualized and quantified by OCT measuring the peripapillary RNFL [43]. The RNFL thickness was measured using the optic disc cube protocol of the Fourier Cirrus HD-OCT (Carl Zeiss Meditec Inc., Dublin, CA, USA) software, version 6.0. This protocol generates a cube of data through a 6-mm square grid by acquiring a series of 200 horizontal scan lines, each composed of 200 A-scans. The RNFL thickness at each pixel is measured and an RNFL thickness map is generated. RNFL thickness quantification is a good measure of axonal integrity associated with VA. It can predict the degree of visual recovery in acute cases of non-MS ON [44,45].

Similarly, macular edema has been related to non-MS ON prognosis [46]. The macular cube 512 × 128 scan was used to obtain MT; this protocol performs 512 horizontal A-scans and 128 vertical B-scan lines within a 6 × 6 mm cube of acquired signal data centered on the fovea. It has been assessed evaluating six areas of the macular cube (superior, superonasal, inferonasal, inferior, inferotemporal and superotemporal sectors) [46]. Besides, MRI was performed to determine optic nerve inflammatory changes and to rule out structural lesions or other causes of ON [47,48,49,50].

Remission was defined as the absence of ON symptoms and signs that lasted longer than 24 h, with or without an associated new lesion on MRI with gadolinium contrast agents for at least 3 months [51,52]. The remission status was classified as complete when there was full recovery of visual outcomes, partial recovery when there was incomplete recovery and no remission when there was no improvement at all [53]. Relapses were defined as new ON symptoms and signs that lasted longer than 24 h, with or without an associated new lesion on MRI with gadolinium contrast agents [52]. AE related to biologic treatment were evaluated and recorded at follow-up.

### 2.3. Data Collection and Statistical Analysis

These outcome variables were recorded in each center according to a follow-up protocol agreed beforehand. Information was stored in a computerized database, and to minimize entry error, all data were double-checked.

Results are expressed as mean ± standard deviation (SD) for the variables with a normal distribution or as median and interquartile range (25th–75th interquartile range (IQR)) for those not normally distributed. Continuous variables were compared with the 2-tailed Student t-test or the Mann–Whitney U-test, as appropriate. The chi-square test or the Fisher exact test was used for the dichotomous variables. The outcome variables were assessed and compared between baseline (at biologic therapy initiation), 1 week, 1 month, 3 months, 6 months and 1 year separately in each outcome, and Wilcoxon signed-rank test was used to assess continuous variables.

## 3. Results

### 3.1. Demographic and Clinical Features at Baseline

We studied 19 patients (11 women/ 8 men) with non-MS ON refractory to systemic glucocorticoids and at least one conventional immunosuppressive drug. The mean age was 34.8 ± 13.9 years. The underlying diseases were idiopathic ON (*n* = 6), Bechet’s disease (*n* = 5), NMO (*n* = 3), systemic lupus erythematosus (SLE) (*n* = 2), sarcoidosis (*n* = 1), relapsing polychondritis (*n* = 1), and myeloperoxidase-anti-neutrophil cytoplasmic antibody-associated vasculitis (*n* = 1). Non-MS ON was unilateral (*n* = 10) and bilateral (*n* = 9). The main demographic and clinical data are summarized in Table 1.

**Figure 1:**
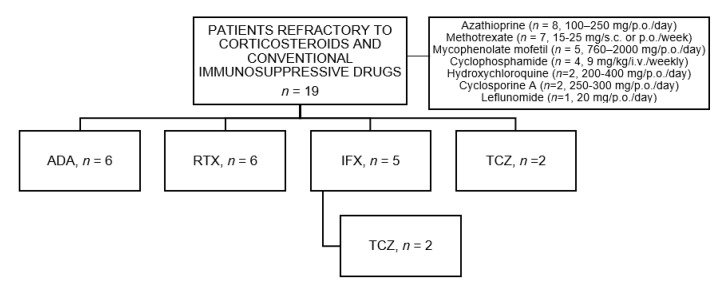
Flow-chart of biologic therapy in refractory non-Multiple Sclerosis optic neuritis. Abbreviations: ADA: Adalimumab, IFX: Infliximab; IS: Immunosuppressive, RTX: Rituximab; TCZ: Tocilizumab.

Before biologic therapy initiation, all patients had received oral glucocorticoids (mean Maximum prednisone dose, 53.7 ± 17.7 mg/day). In 16 cases, i.v. pulses of Methylprednisolone (MP) (mean ± SD dose 3.3 ± 1.5 g) were used before oral glucocorticoids. The conventional immunosuppressive drugs previously used and doses were azathioprine (AZA) (*n* = 8; 100–250 mg/p.o./day), methotrexate (MTX) (*n* = 7; 15–25 mg/s.c. or p.o./week), mycophenolate mofetil (MMF) (*n* = 5; 760–2000 mg/p.o./day), cyclophosphamide (CPM) (*n* = 4; 9 mg/kg/i.v./weekly), hydroxychloroquine (HCQ) (*n* = 2; 200–400 mg/p.o./day), cyclosporine A (CyA) (*n* = 2; 250–300 mg/p.o./day) and leflunomide (LFN) (*n* = 1; 20 mg/p.o/day). (Figure 1).

At biologic therapy initiation, MRI with gadolinium contrast agents was performed in all patients with the following results: normal (*n* = 8), ocular enhancement (chiasma (*n* = 1), choroidal bilateral (*n* = 1), periorbital bilateral (*n* = 1)), spinal cord enhancement (transverse myelitis (*n* = 2); C2-D3 (*n* = 1); C1-T1 (*n* = 1); D5-D7 (*n* = 1)), and cerebral enhancement (frontal subcortical bilateral (*n* = 1), supratentorial (*n* = 1)). CSF fluid analysis was only performed in 3 patients obtaining normal results in all of them.

### 3.2. Biologic Therapy and Efficacy

Biologic agents used were RTX (*n* = 6; two i.v. doses of 1 g/every 2 weeks and then every 6 months), ADA (*n* = 6; 40 mg/sc/1-2 week), IFX (*n* = 5; 5 mg/kg/i.v. at 0, 2 and 6 weeks and then every 8 weeks) and TCZ (*n* = 4; 2 as first and 2 as second biologic therapy; 8 mg/kg/i.v. 2–4 weeks). A second immunosuppressive drug was used simultaneously in 11 patients: MTX (*n* = 11), AZA (*n* = 2), MMF (*n* = 1) and HCQ (*n* = 1). In addition, all patients received oral prednisone (median (IQR) maximum dose at baseline of 40 (10–61.5) mg/day).

**Figure 2:**
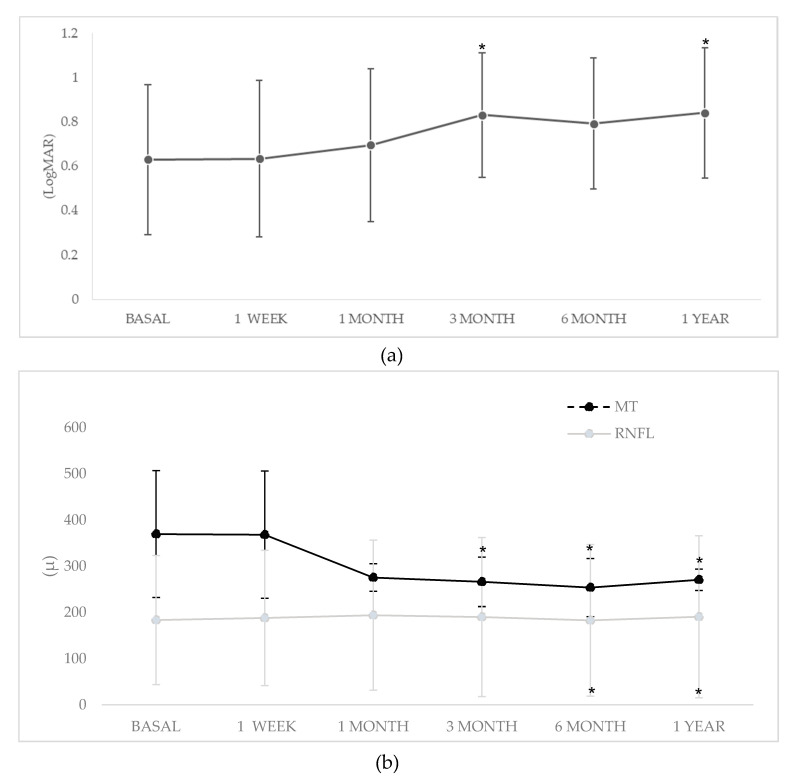
Improvement in (**a**) Best Corrected Visual Acuity (BCVA) and (**b**) Optical Coherence Tomography (OCT) measurements. Abbreviations: BCVA: Best Corrected Visual Acuity; MT: Macular Thickness, OCT: Optical Coherence Tomography; RNFL: Retinal Nerve Fiber Layer. * *p* < 0.05 compared with basal data.

Thus, after one year of biologic therapy, mean ± SD BCVA improved from 0.63 ± 0.34 logMAR to 0.84 ± 0.29 logMAR (*p* = 0.03). Similarly, a significant improvement in optic nerve inflammation was observed since mean ± SD RNFL OCT increased from 183.4 ± 139.6 to 190.5 ± 175.4 µm (*p* = 0.02). Mean ± SD MT decreased from 369.6 ± 137.4 to 270.7 ± 23.2 μm (*p* = 0.03) (Figure 2). A 10.5% (7.1% of eyes) of patients experience a loss in BCVA.

After a mean ± SD follow-up of 35.3 ± 25.1 months, 15 patients (78.9%) achieved ocular remission [51,52]. They were on ADA (*n* = 6, 83.3%), RTX (*n* = 6, 66.6%), IFX (*n* = 5, 60%) and TCZ (*n* = 4, 100%). Only one relapse was described, at 3 months of RTX therapy in one patient.

**Figure 3:**
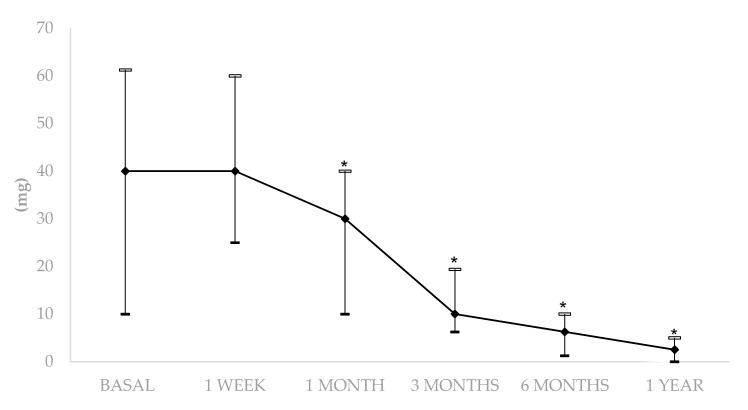
Glucocorticoid sparing effect of biologic therapy in refractory non-Multiple Sclerosis Optic Neuritis. * *p* < 0.05 compared with basal data.

Likewise, a decrease in the median (IQR) prednisone dose was also achieved (40 (10–61.5) mg/day at baseline vs. 2.5 (0–5) mg/day at one year of follow-up; *p* = 0.001) (Figure 3). Before the initiation of biological therapy, all patients required an oral prednisone dose of more than 7.5 mg/day after 3 months. After 3 months of biological therapy, 43.7% of patients required an oral prednisone dose of more than 7.5 mg/day. The biological therapies that required high doses of corticosteroids for more than three months were IFX (*n* = 2), ADA (*n* = 1), RTX (*n* = 1).

Anti-TNFα drugs (ADA, IFX, *n* = 11) were compared with non-anti-TNFα (RTX, TCZ, *n* = 10) agents (Table 2). The underlying diseases were different in both groups. In Bechet’s Disease and sarcoidosis, anti-TNFα were used more frequently, and in NMO and SLE, non-anti-TNFα agents were the most frequently prescribed. At one-month, a greater improvement in BCVA was observed with anti-TNFα drugs (*p* = 0.048) while a greater change from baseline in RNFL OCT was observed with non-anti-TNFα drugs (*p* = 0.007). In any case, at one-year of biologic therapy, improvement in BCVA and RNFL OCT was similar in both groups.

### 3.3. Safety of Biologic Therapy

Severe AEs were observed in 2 patients (10.5%), a 30-year-old woman who suffered a severe infusion-related reaction to RTX and a 28-year-old man who had severe nausea and vomiting while on ADA.

IFX was withdrawn in 2 patients (10.5%) due to ongoing active neuritis. Both developed anti-drug antibodies and tachyphylaxis after 36 and 5 months of treatment. They were switched to TCZ achieving complete remission.

## 4. Discussion

In this study, biologic therapy with both anti-TNFα (ADA, IFX) and non-anti-TNFα (RTX, TCZ) drugs was useful in patients with non-MS ON refractory to systemic glucocorticoids and at least one conventional immunosuppressive drug.

About 20% of patients with non-MS ON are refractory to conventional immunosuppressive drugs [54]. Some studies have analyzed the efficacy of biologic therapy in these cases achieving complete remission in a large number of patients. The use of RTX in NMO has been well-established. Recently, a metanalysis that included 26 studies and 577 participants analyzing the effectiveness of RTX in NMO was conducted [20]. In this study, 62.9% participants reached complete remission. Similarly, in a clinical trial conducted with 7 patients with NMO refractory to treatment, a monthly injection of TCZ was given to all patients. Complete remission was reached in 71.4% of patients. There is less information on anti-TNFα effectiveness. A recent study analyzed the use of IFX in 11 patients with ocular Behçet’s disease. Six patients had ON. A total of 5 patients achieved partial remission, and 1 patient achieved complete remission after a mean ± SD follow-up of 12.3 ± 5.7 years [25]. Two single cases of neurosarcoidosis refractory to treatment achieved partial remission with the use of IFX [55,56]. Recently, other biologic therapies such as satralizumab, eculizumab and inebilizumab have been tested in NMO in three recent clinical trials with promising results [16,17,18]. These new clinical trials underline the necessity of new treatment options for non-MS ON and the importance of biological therapy in management of this disease.

In our study, 15 out of 19 patients were relapse-free, and the treatment response rate was 78.9%. The study showed the efficacy of biologic therapy on refractory non-MS ON and compared the efficacy and safety of four different biologic agents. Patients with idiopathic ON were treated with either anti-TNFα or non-anti TNFα drugs. In contrast, patients with an underlying disease were treated with anti-TNFα or non-anti TNFα agents, based on the underlying disease’s latest treatment guidelines and evidence-based clinical information [57,58,59,60,61,62]. BCVA and RNFL OCT results were different in patients treated with non-anti-TNFα compared to patients treated with anti-TNFα agents in early stages of the study. The reason is unknown. However, it seems that initially a release of large amounts of TNF-α can decrease visual acuity while a high CD19+B cell response and immunoglobulin synthesis could reduce RFNL thickness [63,64]. This initial difference in pathogenesis could maybe explain the disparity of these results.

Biologic therapy was well tolerated in our series. However, two severe AEs related to the use of RTX were observed. Severe AE rate in other studies has been similar to ours: TCZ (1–8%) [65], ADA (5%) [66], IFX (4%) [67], RTX (1–4%) [68]. The slightly higher AEs rate in this study may relate to the smaller sample sizes than in other studies.

The present study has several limitations which affect the generalization of the results. First, the study population was only from Spain. Second, there is an important lack of data in practically every variable. Third, the variability in time of follow-up makes results hard to compare and correlate. A larger scale study should be performed to identify more subtle associations.

In conclusion, the present study suggests that biologic therapy may be effective in patients with non-MS ON refractory to systemic glucocorticoids and conventional immunosuppressive drugs. Further controlled prospective studies with a larger sample are needed to confirm our results. However, patient recruitment might be difficult, as non-MS ON refractory to systemic glucocorticoids and immunosuppressive drugs are a heterogeneous and rare condition. The use of a multicenter international randomized clinical trial would maybe help to overcome not only this challenge but also the challenge that only certain biologics are currently licensed for certain diseases depending on the geographical area, and it would help decrease the cost of these drugs. In the meantime, clinical series are certainly helpful to improve our understanding and management of this disorder.

## Figures and Tables

**Table 1:** Main features of 19 patients with refractory non-Multiple Sclerosis Optic Neuritis treated with biologic therapy.

Case	Gender/Age	Underlying Disease	Cumulative i.v. Steroids Dose (g over 1–3 days)	Max. Oral Prednisone Dose (mg/day)	Conventional IS Drugs	Max. AZA Dose (mg/p.o./day)	Max. MTX Dose (mg/s.c. or p.o./week)	Max. MMF Dose (mg/p.o./day)	Biologic Therapy
1	F/29	Idiopathic	4	60	AZA	150			TCZ
2	F/26	Idiopathic	5.5	30	AZA	100			TCZ
3	F/13	Idiopathic	-	10	MTX		15		ADA
4	F/25	Idiopathic	3	60	MTX		25		IFX, TCZ
5	F/24	Idiopathic	0.5	60	MTX, AZA	100	22.5		ADA
6	M/14	Idiopathic	-	10	MTX		25		ADA
7	M/21	Behçet’s disease	0.5	60	MTX, AZA	150	25		ADA
8	M/25	Behçet’s disease	3	60	MTX, CyA		20		ADA
9	M/39	Behçet’s disease	-	80	MMF			1000	IFX
10	M/40	Behçet’s disease	-	80	MMF			2000	IFX
11	M/37	Behçet’s disease	-	60	CyA				IFX
12	F/68	NMO	2.5	30	CPM, AZA	100			RTX
13	F/41	NMO	3	60	CPM				RTX
14	M/43	NMO	5	60	AZA	250			RTX
15	F/56	SLE	4.5	60	HCQ				RTX
16	F/47	SLE	5	60	HCQ, MMF			750	RTX
17	F/43	Relapsing polychondritis	3	60	MTX, CPM		15		IFX, TCZ
18	M/41	Sarcoidosis	3	40	AZA	100			ADA
19	F/30	Vasculitis ANCA+	3	60	AZA, MMF, LFM, CPM	100		1000	RTX

Abbreviations: ADA: Adalimumab, ANCA: Anti-Neutrophil Cytoplasmic Antibody, AZA: Azathioprine, CPM: Cyclophosphamide, CyA: Cyclosporine A, F: Female, HCQ: Hydroxychloroquine, IS: Immunosuppressive, IFX: Infliximab, i.v. intravenous, IS: Immunosuppressant, M: Male, Max.: Maximum, MMF: Mycophenolate Mofetil, MTX: Methotrexate, NMO: Neuromyelitis Optica RTX: Rituximab, SLE: Systemic Lupus Erythematosus, TCZ: Tocilizumab. Not all patients were available for the planned follow-up at the specified interval. In concrete, there was a loss to follow-up 5 patients after 3 and 6 months. A total of 6 patients were loss to follow-up after 1 year.

**Table 2:** Comparison of patients treated with anti-TNFα and with non-Anti-TNFα.

	Anti-TNFα	Non-Anti-TNFα
*n*	11	8
Sex, *male/female*	7M/4F	1M/7F
Mean age, (*SD*)	29.3 (11.0)	42.5 (14.5)
		
Underlying disease, (*n*)	Behçet’s disease (5) Idiopathic (4) Relapsing polychondritis (1) Sarcoidosis (1)	NMO (3) Idiopathic (2) SLE (2) Vasculitis ANCA+ (1)
		
Conventional IS, (*n*)	MTX (7) AZA (3) MMF (2) CyA (1) CPM (1)	AZA (5) CPM (3) MMF (2) HCQ (2) LFM (1)
		
Second biologic therapy, (*n*)	TCZ (2)	-
Mean follow up in months, (*SD*)	32.6 (20.1)	38.6 (31.4)
Remission, *n* (*%*)	8 (72.7)	5 (62.5)

Abbreviations: ADA: Adalimumab, ANCA: Anti-Neutrophil Cytoplasmic Antibody, AZA: Azathioprine, CPM: Cyclophosphamide, CyA: Cyclosporine A, F: Female, HCQ: Hydroxychloroquine, IFX: Infliximab, IS: Immunosuppressive drug, M: Male, MMF: Mycophenolate Mofetil, MTX: Methotrexate, NMO: Neuromyelitis Optica RTX: Rituximab, SLE: Systemic Lupus Erythematosus, TCZ: Tocilizumab.
